# Reduced thiamine is a predictor for cognitive impairment of cerebral infarction

**DOI:** 10.1002/brb3.1709

**Published:** 2020-08-05

**Authors:** Liang Feng, Weilei He, Guiqian Huang, Shasha Lin, Chengxiang Yuan, Haoran Cheng, Jincai He, Yiming Liu

**Affiliations:** ^1^ Department of Neurology Qilu Hospital Cheeloo College of Medicine Shandong University Jinan China; ^2^ Department of Neurology The First Affiliated Hospital of Wenzhou Medical University Wenzhou China

**Keywords:** cerebral infarction, cognitive impairment, dementia, stroke, thiamine

## Abstract

**Objective:**

Reduced thiamine (vitamin B_1_) had been reported to be associated with cognitive impairment caused by Alzheimer disease. Our study is to explore the association between thiamine and cognitive impairment after acute ischemic stroke.

**Materials and Methods:**

One hundred and eighty two patients with acute cerebral infarction were recruited within the first 24 hr after admission. Thiamine and other vitamin Bs of peripheral blood samples were measured. Patients were divided into with poststroke cognitive impairment (PSCI) and non‐PSCI according to the score of MMSE and the degree of education.

**Results:**

Reduced thiamine (<1.0 ng/ml) was independently associated with PSCI (OR: 2.033, 95% CI: 1.017–4.067, *p* = .045) after adjusting for potential confounding factors. Advanced age, lower education, diabetes mellitus, left hemisphere infarction, and higher National Institute of Health Stroke Scale (NIHSS) were also independent risk factors for PSCI.

**Conclusions:**

Reduced thiamine is one of the predictors for early cognitive impairment in patients with acute cerebral infarction.

## Graphical abstract

PSCI shows a high incidence on acute stage, and more than a half (54.4%) of patients suffered with PSCI. Reduced serum thiamine was associated with early cognitive impairment in patients with acute infarction after adjusting for potential confounding factors. Advanced age, lower education, diabetes mellitus, left hemisphere infarction, and higher NIHSS were also independent risk factors for PSCI.

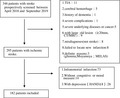

## 1 INTRODUCTION

Poststroke cognitive impairment (PSCI) is a subtype of vascular cognitive impairment (VCI) and one of the important complications along with stroke. Incidence of PSCI ranges from about one‐third to a half within 3 months after stroke (Dichgans & Leys, 2017; Mijajlovic et al., 2017; Nijsse et al., 2017). The etiological factors and precautions for PSCI are being warmly discussed. Many studies showed cognitive impairment resulted from Alzheimer disease (AD) or vascular diseases shared common risk factors, such as diabetes mellitus, atrial fibrillation, and white matter changes(WMCs) (Madhavan, Graff‐Radford, Piccini, & Gersh, 2018; Sachdev et al., 2014; Verdelho et al., 2010). A growing number of studies focus on the relationship between thiamine and dementia. Thiamine insufficiency or functional thiamine deficiency was found to be a character of AD (Gibson et al., 2013, 2016; Pan et al., 2010). As is well known, thiamine is one of the essential vitamins for human beings, and thiamine deficiency is the cause for Wernicke encephalopathy and Korsakoff syndrome. To the best of our knowledge, there was no study which had shed light on the association between thiamine and VCI. The question whether thiamine deficiency is a potential predictor of VCI is attractive and needed to be discovered. PSCI provides a good clinical context for study on VCI. However, mechanisms of PSCI are complex and affected by many different factors. In this study, we collected extensive clinical and neuroimaging data, including basic demographic factors and stroke‐related features. A kind of liquid chromatography–tandem mass spectrometer was used to detect thiamine, together with other vitamin Bs. According to the Diagnostic Criteria for Vascular Cognitive Disorders (A VASCOG Statement) (Sachdev et al., 2014), a new lesion in the brainstem or cerebella is not a responsible change for PSCI, and those with only new subtentorial lesions were excluded from this study. Moreover, the measurement of cognitive function may be affected by depression, and patients with a history of depression or/and poststroke depression (PSD) were also not included in this study (Hommel, Carey, & Jaillard, 2015; Sachdev et al., 2014).

## 2 METHODS

### 2.1 Subjects

Patients with acute ischemic stroke were consecutively recruited from the neurological department of the First Affiliated Hospital of Wenzhou Medical University. This study was approved by the Medical Ethics Committee of the First Affiliated Hospital of Wenzhou Medical University and followed the tenants of the Declaration of Helsinki. The informed consents were signed by the patients and their relatives. The inclusive criteria were listed as follows: (a) an age range from 18 to 90 years old; (b) acute cerebral infarction occurring within 7 days; (c) diagnosed by computerized tomography (CT) or/and magnetic resonance imaging (MRI); (d) the modified Rankin Score (mRS) ≤2 before stroke; and (e) with complete information of regular auxiliary examinations. And the exclusive criteria were listed as follows: (a) transient ischemic attacks (TIA); (b) a history of depression (clinical diagnosis or previous treatment) or/ and PSD, or other psychiatric disorders; (c) unconscious, severe aphasia and dysarthria; (d) with chronic neurological disease, such as dementia, Parkinson's disease (PD), or Parkinson syndrome; (e) with severe underlying diseases; (f) had a history of cancer whether cured or not; (g) failed to complete the measurement of cognition or mood; (h) diagnosed as infratentorial infarction by the neuroimaging; and (i) carrying one or more large old lesions (diameter more than 20 mm) in the imaging.

### 2.2 Medical history

Standardized questionnaires were used in this study and conducted by trained physicians. Demographic data, lifestyle characteristics, and health status of the subjects were collected by the questionnaires within a week after admission. Hypertension was defined if patients had a history of hypertension or were using antihypertensive medication or systolic pressure > 140 mmHg and/or diastolic pressure > 90 mmHg during subject's hospitalization; hyperlipidemia was defined when triglyceride > 1.7 mmol/L, and/or total cholesterol > 5.2 mmol/L, and/or low‐density lipoprotein cholesterol >3.1 mmol/L, or the use of lipid‐lowering drugs. Diabetes mellitus (DM) was defined by a history of DM were using oral hypoglycemic agents/insulin, or/and fasting serum glucose level of 7.0 mmol/L or more, a postprandial serum glucose level of 11.1 mmol/L or more, or/and HbA1c level of 6.5% or more. Ischemic heart disease was defined as a history of myocardial infarction. Atrial fibrillation was diagnosed on a history or the results of electrocardiogram obtained during hospitalization. Anemia was diagnosed according the standard that hemoglobin was <115 g/L for female and 130 g/L for male; hyperuricemia was defined as the level of uric acid above 357 μmol/L for female and 428 μmol/L for male; smoking was defined as current smoking; drinking means drinking regularly (at least once a week), no less than one standard drink (10 g alcohol) a day; and all the patients who had a drinking history were divided into two subgroups according to the amount of drinking: moderate drinking (≤2 standard drink/d) and heavy drinking (>2 standard drink/day). Results within 24 hr after admission of fasting serum glucose (FBG), thyroid hormone (TH), thyrotropin‐releasing hormone (TSH), and homocysteine were also recorded (Table 1).

**TABLE 1:** Baseline clinical characteristics between patients with PSCI and non‐PSCI

	PSCI (*n* = 99)	Non‐PSCI (*n* = 83)	*p*
Age, years	65.30 ± 9.03	61.27 ± 10.17	.001*
Male, *n* (%)	53 (53.5)	59 (71.1)	.015*
Education, years	2 (2–6)	6 (2–8)	.001*
Stroke subtype, *n* (%)			
Large‐artery atherosclerosis, *n*%	32 (32.3)	20 (24.1)	.283
Small‐artery occlusion, *n* (%)	56 (56.6)	56 (67.5)	
Cardioembolism, *n* (%)	8 (8.1)	3 (3.6)	
Other stroke types, *n* (%)	3 (3.0)	4 (4.8)	
Vascular risk factors			
Current smoking, *n* (%)	38 (38.3)	43 (51.8)	.070
Drinking, *n* (%)			
Moderate (≤2 Drink)	11 (11.1)	10 (12.0)	.039*
Heavy (>2 Drink)	13 (13.1)	23 (27.7)	
Hypertension, *n* (%)	65 (65.7)	51 (61.4)	.556
Hyperlipidemia, *n* (%)	65 (65.7)	44 (53.0)	.083
Diabetes mellitus, *n* (%)	48 (48.5)	24 (28.9)	.006*
Prior ischemic TIA or stroke, *n* (%)	15 (15.2)	9 (10.8)	.759
Atrial fibrillation, *n* (%)	11 (11.1)	7 (8.4)	.547
Ischemic heart disease, *n* (%)	4 (4.0)	2 (2.4)	.539
NIHSS	3 (2–6)	2 (1–4)	.001*
HAMA, *n* (%)	10 (10.1)	4 (4.8)	.183
MMSE	13.79 ± 5.02	23.82 ± 3.09	.000*
Results of regular blood test			
WBC	6.52 (5.55–7.70)	6.18 (5.31–7.59)	.333
RBC	4.48 ± 0.44	4.55 ± 0.47	.299
Anemia	10 (10.1)	6 (7.2)	.496
Plt	227.32 ± 54.68	219.14 ± 57.10	.326

Note

Values are means (standard deviations) in Student's *t* test or medians (interquartile range) in Mann–Whitney U test for continuous variables. Values are *n* (%) for categorical variables in chi‐square test. *p* value is the result of statistical comparison between PSCI and non‐PSCI.

* *p* < .05.

### 2.3 Variables of neuroimaging

Brain imaging (CT and/or MRI) after stroke was obtained. An acute infarct was defined by the presence of a hyperintense MRI DWI lesion or/and hypodense lesions on CT that were relevant to the acute neurological symptoms. A radiologist and a neurologist both viewed the neuroimagings, and results were got with agreement. The following informations were recorded: size, location, and laterality of lesions. Infarcts were categorized as small if the diameter ≤20 mm (divided as single or multiple) or large if the diameter >20 mm. Locations of infarct were classified into cortical, subcortical white matter, and deep (basal ganglia, internal capsule, or thalamus). The lesion was labeled as left or right hemisphere of the brain. White matter lesion was evaluated with Fazekas scale (from 0 to 3 point) according to the degree (Defrancesco et al., 2013; Heo et al., 2009). Ischemic stroke was classified into large‐artery atherosclerosis, small‐artery occlusion, cardioembolism, and stroke of other cause (more than two causes or undetermined causes), according to the TOAST criteria (Trial of Org 10172 in Acute Stroke Treatment) (Adams et al., 1993), with exception that acute small infarct using the upper limit of 20 mm according to the up‐to‐date studies (Sachdev et al., 2014; Wardlaw et al., 2013; Yang et al., 2015).

### 2.4 Scale measurement and definition of PSCI

The following scale measurement was used for measurement in this study: the National Institutes of Health Stroke Scale (NIHSS), which was performed by trained physicians who in charge of the patients at admission; the Chinese version of Mini‐Mental State Examination (MMSE),17‐item Hamilton Depression Rating Scale (HAMD); and the 17‐item Hamilton Anxiety Rating Scale (HAMA), which were performed by only one fixed psychiatrist blind to all the clinical massages of the patients. Poststroke depression (PSD) was defined when HAMD score > 7 points, and those with PSD were finally excluded in this study; anxiety symptoms were screened by HAMA, and poststroke anxiety (PSA) was defined when HAMA score > 7 points. The PSCI was diagnosed by the MMSE scores and adjusted according to education years. Patients who were illiterate according the level ≤17 points, and a primary school education were according the level ≤ 20 points, postsecondary education or above were according the level ≤ 24 points (Cui et al., 2011; Katzman et al., 1988; Yao et al., 2010).

### 2.5 Blood collection and testing of vitamin Bs

Serum samples were obtained by centrifugation in the next morning after admission. Samples for the determination of vitamin Bs were stored at −80°C before being assayed. Thiamine and other vitamin Bs (vitamins B_2_, B_3_, B_5_, and B_9_) were measured using a kind of liquid chromatography–tandem mass spectrometer (Triple QuadTM 4500MD System, Sciex). A laboratory technician blinded to all clinical data processed the samples. The normal reference values of vitamin Bs were listed as follows: Thiamine was 1.0–10.10 ng/ml, vitamin B_2_ was 2.30–14.60 ng/ml, vitamin B_3 _was 5.2–72.10 ng/ml, vitamin B_5_ was12.90–253.10 ng/ml, and vitamin B_9_ was above 3.0 ng/ml. For results of blood tests, see Table 3.

### 2.6 Statistical analyses

To continuous variables on normal distribution, results were expressed as the mean ± standard deviation (*SD*) and compared using Student's *t* test; while data on normal distribution, variables were exhibited as median (interquartile range) and compared using the Mann–Whitney *U* test. Categorical variables were listed as number (percentage) and compared using the chi‐squared test. The significant variables including thiamine were further taken into logistic regression analysis and adjusted for the potential confounding factors. In logistic regression analysis, education was dichotomized as lower education (≤3 years, by median), and NIHSS was dichotomized as severity (≥4 point, by median); drinking was divided into three groups (no drinking, moderate, and heavy). The results of logistic regression analysis were expressed as adjusted odds ratios (OR) with the corresponding 95% confidence intervals (CI). Software of SPSS19.0 was adopted for statistical analysis. Values of *p* < .05 were considered to be statistically significant in all tests.

## 3 RESULTS

**FIGURE 1:**
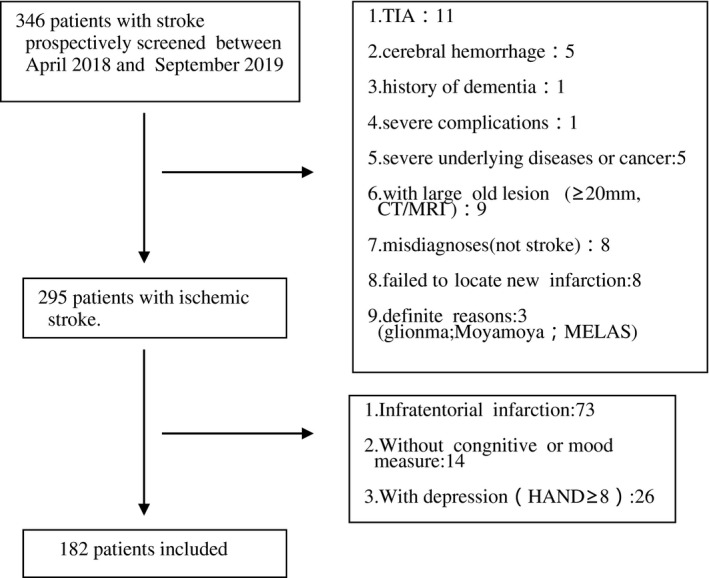
The screening process of subjects in this study

Between April 2018 and September 2019, 356 admitted patients with acute ischemic stroke in all were screened, and 182 patients were included in our study (for the screening process of subjects, see Figure 1). In this study, 99 patients (54.4%) were diagnosed with PSCI on acute stage, while 83 patients (46.4%) without PSCI. The baseline information of the two groups is displayed in Table 1. Compared to non‐PSCI, patients with PSCI were significantly older (*p* = .001) and had higher NIHSS (*p* = .001), lower education (*p* = .001), and higher proportion of diabetes mellitus (*p* = .006) (Table 1)and left hemisphere infarction (*p* = .005) (Table 2). Besides, low level of thiamine was more often in patients with PSCI than those without PSCI (*p* = .011) (Table 3).Variables of male (*p* = .015) and drinking history (*p* = .039) were also considered to be statistically significant in analysis of single factor (Table 1).

**TABLE 2:** Neuroimaging features in patients between PSCI and non‐PSCI

	PSCI (*n* = 99)	Non‐PSCI (*n* = 83)	*p*
Acute ischemic lesions			
Single small infarct(size ≤ 20 mm)	46	51	.130
Multiple small infarct (*n* ≥ 2, size ≤20 mm)	22	13	
Large infarct (*n* ≥ 1, size >20 mm)	31	19	
Location of acute infarction (%)			
Cortical	15	14	.690
Subcortical white matter	43	32	
Deep	22	24	
More than two regions	19	13	
Lateralization, *n* (%)			
Left hemisphere	60	33	.005*
Right hemisphere	39	50	
WM lesion(by Fazekas scale)			
Grade0	14	10	.100
Grade1	48	38	
Grade2	25	26	
Grade3	16	5	

Note

Values are *n* (%) for categorical variables in chi‐square test. *p* value is the result of statistical comparison between PSCI and non‐PSCI.

* *p* < .05.

**TABLE 3:** Blood test results of vitamin Bs between PSCI and non‐PSCI

	PSCI (*n* = 99)	Non‐PSCI (*n* = 83)	*p*
VB1 (<1 ng/ml), *n* (%)	52 (52.5)	30 (36.1)	.011*
VB2, ng/ml	10.90 (1.40–55.90)	12.50 (3.70–76.60)	.110
VB3, ng/ml	25.60 (7.20–112.00)	30.80 (19.80–42.00)	.141
VB5, ng/ml	40.79 ± 17.21	41.57 ± 17.86	.933
VB9 (<3 ng/ml), *n* (%)	29 (29.3)	33 (39.8)	.285

Note

Values are means (standard deviations) in Student's *t* test or medians (interquartile range) in Mann–Whitney U test for continuous variables. Values are *n* (%) for categorical variables in chi‐square test. *p* value is the result of statistical comparison between PSCI and non‐PSCI.

* *p* < .05.

There were no significant group differences in terms of stroke subtype, size of ischemic lesion, white matter lesion (Table 2), and other vascular risk factors, such as current smoking, hypertension, hyperlipidemia, ischemic heart disease, and atrial fibrillation (Table 1). Meanwhile, there was no significant difference in variables of anemia, hyperuricemia (HUA), fasting serum glucose (FBG), thyroid hormone (TH), thyrotropin‐releasing hormone (TSH), homocysteine (HCY) (Table 1), and other vitamin Bs between the two groups (Table 3).

In a binary logistic regression, reduced thiamine was independently associated with the development of PSCI (OR: 2.033, 95% CI: 1.017–4.067, *p* = .045). Advanced age (OR: 1.053, 95% CI: 1.014–1.094, *p* = .008), lower education (OR: 2.144, 95% CI: 1.034–4.447, *p* = .040), diabetes mellitus (OR: 2.433, 95% CI: 1.192–4.967, *p* = .015), infarction in left hemisphere (OR: 2.699 95% CI: 1.349–5.398, *p* = .005), and higher NIHSS (OR: 3.081, 95% CI: 1.478–6.425, *p* = .003) were also independent risk factors of PSCI in this study (Table 4).

**TABLE 4:** Results of multivariate logistic regression for the features associated with PSCI

Variables	OR	95% CI	*p* value
Age, years	1.053	1.014–1.094	.008*
Male	0.781	0.344–1.772	.554
Drinking	0.749	0.456–1.229	.252
Diabetes mellitus	2.433	1.192–4.967	.015*
Left hemisphere infarction	2.699	1.349–5.398	.005*
Higher NIHSS (≥4)	3.081	1.478–6.425	.003*
Lower education (≤3 years)	2.144	1.034–4.447	.040*
Lower thiamine (<1 ng/ml)	2.033	1.017–4.067	.045*

Note

*p* value is the result of statistical comparison by multivariate logistic regression between PSCI and non‐PSCI.

* *p* < .05.

## 4 DISCUSSION

In our study, among 182 patients, more than a half (54.4%) were diagnosed with PSCI on the acute stage of cerebral infarction. Low level of thiamine is independently associated with early cognitive impairment (OR: 2.033, 95% CI: 1.017–4.067, *p* = .045), after adjusted for confounding factors. To the best of our knowledge, this is the first research to explore the relationship between thiamine and PSCI on acute stage.

As we know, glucose metabolism is a basic vital movement for human being. There are four forms of thiamin which exist in humans: unphosphorylated thiamine, thiamine monophosphate, thiamine diphosphate, and thiamine triphosphate. Thiamine diphosphate (TDP), which also named thiamine pyrophosphate (TPP), is the biologically active form of thiamine. The brain uses 25% of the glucose with only 2% of body weight in awake state (Chen & Zhong, 2013). TDP acts at key steps of glucose metabolism: In the pentose shunt, TDP is a coenzyme of transketolase; in the tricarboxylic acid (TCA) cycle, TDP is a coenzyme both for pyruvate dehydrogenase complex (PDHC) and ketoglutarate dehydrogenase complex (KGDHC) (Chen & Zhong, 2013; Gibson & Blass, 2007; Gibson et al., 2013, 2016);thus, thiamine deficiency would interfere the metabolism of glucose. Meanwhile, the decrease of glucose metabolism in the brain is a characteristic change for dementia and can be detected by 18F‐fluorodeoxyglucose (FDG)‐PET (Chen & Zhong, 2013; Leon et al., 2001; Ossenkoppele et al., 2013; Small et al., 2008).The mechanism underlying the association between reduced thiamine and PSCI may be explained as follows: On one side, it seemed reasonable that low level of thiamine connects with the decrease of glucose metabolism in central nervous system (CNS), which may lead to impairment of cognition. On the other hand, acute cerebral infarction in the brain, especially in the strategic location, would cause metabolism disorder, including a functional thiamine deficiency (Biesbroek, Weaver, & Biessels, 2017), which may do more harm to cognitive impairment. Moreover, it is usually unviable to estimate the level of thiamine together with the cognition function before stroke, and the accurate period of thiamine deficiency is unknown. A chronic deficiency of thiamine may first change the function of endothelial cells in CNS; other cell types, like astrocytes and microglias, will be involved as the state going on, and then, chronic inflammation and oxidative stress may take place in CNS (Gibson et al., 2016). Studies show that thiamine acts as an antioxidant and thiamine deficiency (TD) exacerbates the plaque pathology and increases the phosphorylation of tau in mouse model (Karuppagounder et al., 2009).Thiamine was found binded to multiple mitochondrial enzymes and may alter the interaction of the mitochondria and TD may cause endoplasmic reticulum (ER) stress, and autophagy in the brain (Liu, Ke, & Luo, 2017; Mkrtchyan et al., 2015). Thiamine deficiency (TD) also reduces activity of choline acetyltransferase (ChAT) which will cause the decrease of acetylcholine; TD also induces excess glutamate release, both of these are important pathogenesis for dementia. Furthermore, in animal models, TD reduces neurogenesis, especially in thalamus, hippocampus, and prefrontal cortex, and these change take place before the morphological alteration in the brain (Gibson et al., 2016; Zhao et al., 2008). Chronic deficiency of thiamine may coexist with those risk factors for VCI and exacerbate cognitive impairment in high‐risk population.

Nevertheless, what is the reason for the decrease of thiamine？ It seemed that thiamine deficiency is common in ordinary people, especially among elderly people and those with chronic disease (Borg et al., 2015; Hoffman, 2016). In our study, the following may explain thiamine deficiency in patients with stroke: First, insufficiency of intake, personal dietary choices before or after stroke may lead to thiamine insufficiency; besides a chronic low intake, metatrophia may occur after stroke. Second, physical condition of patients affected on the absorb and storage of thiamine in the body. Chronic diseases such as diabetes, cardiac failure, and gastrointestinal diseases could lead to an apparent thiamine deficiency (Gibson et al., 2016; Pepersack et al., 1999; Thornalley et al., 2007). Third, many different drugs can influence the thiamine status, including antacids (proton pump inhibitors), antihypertensives, diuretics, and antidepressants (Gibson et al., 2016). Although patients with a history of depression or PSD and those with severe comorbidity were excluded in our study, other risk factors such as diabetes, using antihypertensives or proton pump inhibitors were common among patients with ischemic stroke.

In the present study, we also found that advanced age, lower education, diabetes mellitus, left hemisphere infarction, and higher NIHSS were independently associated with early cognitive impairment. Aging was the most common risk factor for cognitive impairment, while education was a protective factor (Chen et al., 2016; Farooq & Gorelick, 2013; Yu et al., 2013). Although the entry criterias were variable, diabetes mellitus seemed to be the most common vascular risk factor for cognitive impairment and was a predictor of cognitive impairment after stroke (Ding, Xu, & Wang, 2019; Swardfager & MacIntosh, 2017; Wang et al., 2018). Strategic subcortical infarcts including internal capsule, thalamus, fornix, and caudate nucleus were the causes for PSCI (Biesbroek et al., 2017; Sachdev et al., 2014); though the lesions of infarction could not be defined in detail in most studies including ours, patients showed different cognitive impairment on left or right hemisphere. Left hemisphere was usually to be considered as dominant hemisphere, and infarction on left hemisphere was more easily to develop into cognitive disorder. The result was in accord with neuropsychological function of dominant hemisphere. Higher NIHSS means more neurological function defective after stroke, which was connected with cognitive impairment during both acute stage and chronic rehabilitation period (Chang et al., 2017).

There were still some limitations in our study. First, cognitive function was assessed on acute stage, and it is necessary to investigate the development of cognitive function during the long‐term follow‐up. Second, we excluded patients with depression, severe aphasia, and serious comorbidity, which might lead to an underestimation of the actual incidence of PSCI. Third, the level of thiamine was measured only once (at admission), and our future study will be conducted to measure thiamine dynamically. Last, the sample size of this study is not so large.

## 5 CONCLUSION

Poststroke cognitive impairment shows a high incidence on acute stage, and more than a half (54.4%) of patients suffered with PSCI. Reduced serum thiamine was associated with early cognitive impairment in patients with acute infarction after adjusting for potential confounding factors. Advanced age, lower education, diabetes mellitus, left hemisphere infarction, and higher NIHSS were also independent risk factors for PSCI.

## CONFLICTS OF INTEREST

All the authors declare that there are no conflicts of interest.

## AUTHOR CONTRIBUTIONS

Yiming Liu and Jincai He conceived and designed the experiments. Liang Feng drafted and revised the article and analyzed and interpreted the data. Weilei He nd Guiqian Huang screened the subjects and collected the basic data. Shasha Lin, Chengxiang Yuan, and Haoran Cheng collected and stored the blood samples.All authors read and approved the final manuscript.

### PEER REVIEW

The peer review history for this article is available at https://publons.com/publon/10.1002/brb3.1709.

[Correction added on September 9,2020 , after first online publication: Peer review history statement has been added.]

## Data Availability

All data generated or analyzed during this study are included in this article.

## References

[brb31709-bib-0001] Adams Jr, H. P. , Bendixen, B. H. , Kappelle, L. J. , Biller, J. , Love, B. B. , Gordon, D. L. , & Marsh, E. E. (1993). Classification of subtype of acute ischemic stroke. Definitions for use in a multicenter clinical trial. TOAST. Trial of Org 10172 in Acute Stroke Treatment. Stroke, 24(1), 35–41. 10.1161/01.STR.24.1.35 7678184

[brb31709-bib-0002] Biesbroek, J. M. , Weaver, N. A. , & Biessels, G. J. (2017). Lesion location and cognitive impact of cerebral small vessel disease. Clinical Science, 131(8), 715–728. 10.1042/CS20160452 28385827

[brb31709-bib-0003] Chang, W. H. , Sohn, M. K. , Lee, J. , Kim, D. Y. , Lee, S.‐G. , Shin, Y. , … Kim, Y.‐H. (2017). Long‐term functional outcomes of patients with very mild stroke: Does a NIHSS score of 0 mean no disability? An interim analysis of the KOSCO study. Disability and Rehabilitation, 39(9), 904–910. 10.3109/09638288.2016.1170214 27206550

[brb31709-bib-0004] Chen, X. , Duan, L. , Han, Y. , Tian, L. , Dai, Q. , Wang, S. , … Liu, X. (2016). Predictors for vascular cognitive impairment in stroke patients. BMC Neurology, 16, 115 10.1186/s12883-016-0638-8 27461245 PMC4962370

[brb31709-bib-0005] Chen, Z. , & Zhong, C. (2013). Decoding Alzheimer's disease from perturbed cerebral glucose metabolism: Implications for diagnostic and therapeutic strategies. Progress in Neurobiology, 108, 21–43. 10.1016/j.pneurobio.2013.06.004 23850509

[brb31709-bib-0006] Cui, G. H. , Yao, Y. H. , Xu, R. F. , Tang, H.‐D. , Jiang, G.‐X. , Wang, Y. , … Cheng, Q. (2011). Cognitive impairment using education‐based cutoff points for CMMSE scores in elderly Chinese people of agricultural and rural Shanghai China. Acta Neurologica Scandinavica, 124(6), 361–367. 10.1111/j.1600-0404.2010.01484.x 21303351

[brb31709-bib-0007] de Leon, M. J. , Convit, A. , Wolf, O. T. , DeSanti, S. , Rusinek, H. , Tsui, W. , … Thorn, E. (2001). Prediction of cognitive decline in normal elderly subjects with 2‐[(18)F]fluoro‐2‐deoxy‐D‐glucose/poitron‐emission tomography (FDG/PET). Proceedings of the National Academy of Sciences of the United States of America, 98(19), 10966–10971. 11526211 10.1073/pnas.191044198 PMC58582

[brb31709-bib-0008] Defrancesco, M. , Marksteiner, J. , Deisenhammer, E. , Kemmler, G. , Djurdjevic, T. , & Schocke, M. (2013). Impact of white matter lesions and cognitive deficits on conversion from mild cognitive impairment to Alzheimer's disease. Journal of Alzheimer's Disease: JAD, 34(3), 665–672. 10.3233/JAD-122095 23254639

[brb31709-bib-0009] Dichgans, M. , & Leys, D. (2017). Vascular cognitive impairment. Circulation Research, 120(3), 573–591. 10.1161/CIRCRESAHA.116.308426 28154105

[brb31709-bib-0010] Ding, M. Y. , Xu, Y. , Wang, Y. Z. et al (2019). Predictors of cognitive impairment after stroke: A prospective stroke cohort study. Journal of Alzheimer's Disease, 71(4), 1139–1151. 10.3233/JAD-190382 31524163

[brb31709-bib-0011] Farooq, M. U. , & Gorelick, P. B. (2013). Vascular cognitive impairment. Current Atherosclerosis Reports, 15(6), 330 10.1007/s11883-013-0330-z 23612956

[brb31709-bib-0012] Gibson, G. E. , & Blass, J. P. (2007). Thiamine‐dependent processes and treatment strategies in neurodegeneration. Antioxidants & Redox Signaling, 9(10), 1605–1619. 10.1089/ars.2007.1766 17685850

[brb31709-bib-0013] Gibson, G. E. , Hirsch, J. A. , Cirio, R. T. , Jordan, B. D. , Fonzetti, P. , & Elder, J. (2013). Abnormal thiamine‐dependent processes in Alzheimer's Disease. Lessons from diabetes. Molecular and Cellular Neuroscience, 55, 17–25. 10.1016/j.mcn.2012.09.001 22982063 PMC3609887

[brb31709-bib-0014] Gibson, G. E. , Hirsch, J. A. , Fonzetti, P. , Jordan, B. D. , Cirio, R. T. , & Elder, J. (2016). Vitamin B1 (thiamine) and dementia. Annals of the New York Academy of Sciences, 1367(1), 21–30. 10.1111/nyas.13031 26971083 PMC4846521

[brb31709-bib-0015] Heo, J. H. , Lee, S. T. , Kon, C. , Park, H. J. , Shim, J. Y. , & Kim, M. (2009). White matter hyperintensities and cognitive dysfunction in Alzheimer disease. Journal of Geriatric Psychiatry and Neurology, 22(3), 207–212. 10.1177/0891988709335800 19433863

[brb31709-bib-0016] Hoffman, R. (2016). Thiamine deficiency in the Western diet and dementia risk. The British Journal of Nutrition, 116(1), 188–189. 10.1017/S000711451600177X 27170224

[brb31709-bib-0017] Hommel, M. , Carey, L. , & Jaillard, A. (2015). Depression: Cognition relations after stroke. International Journal of Stroke, 10(6), 893–896. 10.1111/ijs.12057 24165205

[brb31709-bib-0018] Karuppagounder, S. S. , Xu, H. , Shi, Q. , Chen, L. H. , Pedrini, S. , Pechman, D. , … Gibson, G. E. (2009). Thiamine deficiency induces oxidative stress and exacerbates the plaque pathology in Alzheimer's mouse model. Neurobiology of Aging, 30(10), 1587–1600. 10.1016/j.neurobiolaging.2007.12.013 18406011 PMC2782730

[brb31709-bib-0019] Katzman, R. , Zhang, M. Y. , Ouang Ya, Q. , Wang, Z. , Liu, W. T. , Yu, E. , … Grant, I. (1988). A Chinese version of the Mini‐Mental State Examination; impact of illiteracy in a Shanghai dementia survey. Journal of Clinical Epidemiology, 41(10), 971–978. 10.1016/0895-4356(88)90034-0 3193141

[brb31709-bib-0020] Liu, D. , Ke, Z. , & Luo, J. (2017). Thiamine deficiency and neurodegeneration: The interplay among oxidative stress, endoplasmic reticulum stress, and autophagy. Molecular Neurobiology, 54(7), 5440–5448. 10.1007/s12035-016-0079-9 27596507 PMC5337452

[brb31709-bib-0021] Madhavan, M. , Graff‐Radford, J. , Piccini, J. P. , & Gersh, B. J. (2018). Cognitive dysfunction in atrial fibrillation. Nature Reviews Cardiology, 15(12), 744–756. 10.1038/s41569-018-0075-z 30275499

[brb31709-bib-0022] Mijajlovic, M. D. , Pavlovic, A. , Brainin, M. , Heiss, W.‐D. , Quinn, T. J. , Ihle‐Hansen, H. B. , … Bornstein, N. M. (2017). Post‐stroke dementia ‐ a comprehensive review. BMC Medicine., 15(1), 11 10.1186/s12916-017-0779-7 28095900 PMC5241961

[brb31709-bib-0023] Mkrtchyan, G. , Aleshin, V. , Parkhomenko, Y. , Kaehne, T. , Luigi Di Salvo, M. , Parroni, A. , … Bunik, V. (2015). Molecular mechanisms of the non‐coenzyme action of thiamin in brain: Biochemical, structural and pathway analysis. Scientific Reports, 5, 12583 10.1038/srep12583 26212886 PMC4515825

[brb31709-bib-0024] Nijsse, B. , Visser‐Meily, J. M. , van Mierlo, M. L. , Post, M. W. , de Kort, P. L. , & van Heugten, C. M. (2017). Temporal evolution of poststroke cognitive impairment using the montreal cognitive assessment. Stroke, 48(1), 98–104. 10.1161/STROKEAHA.116.014168 27899753

[brb31709-bib-0025] Ossenkoppele, R. , Prins, N. D. , Pijnenburg, Y. A. , Lemstra, A. W. , van der Flier, W. M. , Adriaanse, S. F. , … van Berckel, B. N. M. (2013). Impact of molecular imaging on the diagnostic process in a memory clinic. Alzheimer's & Dementia: The Journal of the Alzheimer's Association, 9(4), 414–421. 10.1016/j.jalz.2012.07.003 23164552

[brb31709-bib-0026] Pan, X. , Gong, N. , Zhao, J. , Yu, Z. , Gu, F. , Chen, J. , … Dong, W. (2010). Powerful beneficial effects of benfotiamine on cognitive impairment and beta‐amyloid deposition in amyloid precursor protein/presenilin‐1 transgenic mice. Brain: A Journal of Neurology, 133(Pt 5), 1342–1351. 20385653 10.1093/brain/awq069

[brb31709-bib-0027] Pepersack, T. , Garbusinski, J. , Robberecht, J. , Beyer, I. , Willems, D. , & Fuss, M. (1999). Clinical relevance of thiamine status amongst hospitalized elderly patients. Gerontology, 45(2), 96–101. 10.1159/000022070 9933732

[brb31709-bib-0028] Sachdev, P. , Kalaria, R. , O'Brien, J. , Skoog, I. , Alladi, S. , Black, S. E. , … Scheltens, P. (2014). Diagnostic criteria for vascular cognitive disorders: A VASCOG statement. Alzheimer Disease and Associated Disorders, 28(3), 206–218. 10.1097/WAD.0000000000000034 24632990 PMC4139434

[brb31709-bib-0029] Small, G. W. , Bookheimer, S. Y. , Thompson, P. M. , Cole, G. M. , Huang, S.‐C. , Kepe, V. , & Barrio, J. R. (2008). Current and future uses of neuroimaging for cognitively impaired patients. The Lancet Neurology, 7(2), 161–172. 10.1016/S1474-4422(08)70019-X 18207114 PMC2728702

[brb31709-bib-0030] Swardfager, W. , & MacIntosh, B. J. (2017). Depression, type 2 diabetes, and poststroke cognitive impairment. Neurorehabilitation and Neural Repair, 31(1), 48–55. 10.1177/1545968316656054 27364648

[brb31709-bib-0031] ter Borg, S. , Verlaan, S. , Hemsworth, J. , Mijnarends, D. M. , Schols, J. M. G. A. , Luiking, Y. C. , & de Groot, L. C. P. G. M. (2015). Micronutrient intakes and potential inadequacies of community‐dwelling older adults: A systematic review. The British Journal of Nutrition, 113(8), 1195–1206. 10.1017/S0007114515000203 25822905 PMC4531469

[brb31709-bib-0032] Thornalley, P. J. , Babaei‐Jadidi, R. , Al Ali, H. , Rabbani, N. , Antonysunil, A. , Larkin, J. , … Bodmer, C. W. (2007). High prevalence of low plasma thiamine concentration in diabetes linked to a marker of vascular disease. Diabetologia, 50(10), 2164–2170. 10.1007/s00125-007-0771-4 17676306 PMC1998885

[brb31709-bib-0033] Verdelho, A. , Madureira, S. , Moleiro, C. , Ferro, J. M. , Santos, C. O. , Erkinjuntti, T. , … Inzitari, D. (2010). White matter changes and diabetes predict cognitive decline in the elderly: The LADIS study. Neurology, 75(2), 160–167. 10.1212/WNL.0b013e3181e7ca05 20625169

[brb31709-bib-0034] Wang, Q. , Zhao, K. , Cai, Y. , Tu, X. , Liu, Y. , & He, J. (2018). Prediabetes is associated with post‐stroke cognitive impairment in ischaemic stroke patients. Brain Research, 1687, 137–143. 10.1016/j.brainres.2017.12.034 29289546

[brb31709-bib-0035] Wardlaw, J. M. , Smith, E. E. , Biessels, G. J. , Cordonnier, C. , Fazekas, F. , Frayne, R. , … Dichgans, M. (2013). Neuroimaging standards for research into small vessel disease and its contribution to ageing and neurodegeneration. The Lancet Neurology, 12(8), 822–838. 10.1016/S1474-4422(13)70124-8 23867200 PMC3714437

[brb31709-bib-0036] Yang, J. , Wong, A. , Wang, Z. , Liu, W. , Au, L. , Xiong, Y. , … Mok, V. C. T. (2015). Risk factors for incident dementia after stroke and transient ischemic attack. Alzheimer's & Dementia: The Journal of the Alzheimer's Association, 11(1), 16–23. 10.1016/j.jalz.2014.01.003 24603162

[brb31709-bib-0037] Yao, Y. H. , Xu, R. F. , Tang, H. D. , Jiang, G.‐X. , Wang, Y. , Wang, G. , … Cheng, Q. I. . (2010). Cognitive impairment and associated factors among the elderly in the Shanghai suburb: Findings from a low‐education population. Neuroepidemiology, 34(4), 245–252. 10.1159/000297751 20299806

[brb31709-bib-0038] Yu, K. H. , Cho, S. J. , Oh, M. S. , Jung, S. , Lee, J.‐H. , Shin, J.‐H. , … Lee, B.‐C. (2013). Cognitive impairment evaluated with vascular cognitive impairment harmonization standards in a multicenter prospective stroke cohort in Korea. Stroke, 44(3), 786–788. 10.1161/STROKEAHA.112.668343 23271507

[brb31709-bib-0039] Zhao, N. , Zhong, C. , Wang, Y. , Zhao, Y. , Gong, N. , Zhou, G. , … Hong, Z. (2008). Impaired hippocampal neurogenesis is involved in cognitive dysfunction induced by thiamine deficiency at early pre‐pathological lesion stage. Neurobiology of Disease, 29(2), 176–185. 10.1016/j.nbd.2007.08.014 17936635

